# RETRACTION: Effects of Refined Nursing Interventions in the Operating Room on Surgical‐Site Wound Infection in Patients With Lung Cancer: A Meta‐Analysis

**DOI:** 10.1111/iwj.70319

**Published:** 2025-03-06

## Abstract

**RETRACTION:** WangX. , LiX. , and ZhangJ. , “Effects of Refined Nursing Interventions in the Operating Room on Surgical‐Site Wound Infection in Patients With Lung Cancer: A Meta‐Analysis,” International Wound Journal 21, no. 2 (2024): e14391, 10.1111/iwj.14391.PMC1082812337743559

The above article, published online on 24 September 2023, in Wiley Online Library (http://onlinelibrary.wiley.com/), has been retracted by agreement between the journal Editor in Chief, Professor Keith Harding; and John Wiley & Sons Ltd. Following an investigation by the publisher, all parties have concluded that this article was accepted solely on the basis of a compromised peer review process. The editors have therefore decided to retract the article. The authors did not respond to our notice regarding the retraction.

**RETRACTION:** X. Wang , X. Li , and J. Zhang , “Effects of Refined Nursing Interventions in the Operating Room on Surgical‐Site Wound Infection in Patients With Lung Cancer: A Meta‐Analysis,” International Wound Journal 21, no. 2 (2024): e14391, 10.1111/iwj.14391.PMC1082812337743559

The above article, published online on 24 September 2023, in Wiley Online Library (http://onlinelibrary.wiley.com/), has been retracted by agreement between the journal Editor in Chief, Professor Keith Harding; and John Wiley & Sons Ltd. Following an investigation by the publisher, all parties have concluded that this article was accepted solely on the basis of a compromised peer review process. The editors have therefore decided to retract the article. The authors did not respond to our notice regarding the retraction.

